# Psychosocial Adjustment Changes and Related Factors in Postoperative Oral Cancer Patients: A Longitudinal Study

**DOI:** 10.3390/biomedicines10123231

**Published:** 2022-12-12

**Authors:** Yi-Wei Chen, Ting-Ru Lin, Pei-Ling Kuo, Shu-Chiung Lee, Kuo-Feng Wu, Tuyen Van Duong, Tsae-Jyy Wang

**Affiliations:** 1 School of Nursing, National Taipei University of Nursing and Health Sciences, Taipei City 112, Taiwan; 2 Department of Cardiothoracic Surgery, Hualien Tzu Chi Hospital, Buddhist Tzu Chi Medical Foundation, Hualien City 970, Taiwan; 3 Department of Nursing, Cardinal Tien College of Healthcare and Management, New Taipei City 231, Taiwan; 4 School of Nursing, National Yang-Ming Chiao Tung University, Taipei City 112, Taiwan; 5 Department of Nursing, Taipei Veterans General Hospital, Taipei City 112, Taiwan; 6 Department of Nurse-Midwifery and Women Health, National Taipei University of Nursing and Health Science, Taipei City 112, Taiwan; 7 School of Nutrition & Health Sciences, Taipei Medical University, Taipei City 110, Taiwan; 8 International Master/Ph.D. Program in Medicine, College of Medicine, Taipei Medical University, Taipei City 110, Taiwan

**Keywords:** psychosocial adjustment, oral cancer, symptoms, facial disfigurement

## Abstract

Disease and treatment-related symptoms and dysfunctions can interfere with the psychosocial adjustment of patients with oral cancer. Identifying factors influencing psychosocial maladjustment is important because at-risk individuals can be targeted for early intervention. This prospective longitudinal study investigated psychosocial adjustment changes and associated factors in postoperative oral cancer patients. Data on psychosocial adjustment, facial disfigurement, symptoms, and social support were collected before surgery (T1) at one month (T2), three months (T3), and five months after discharge (T4). Fifty subjects completed the study, and their data were included in the analysis. Psychosocial maladjustment was reported in 50%, 59.2%, 66%, and 62% of subjects at T1, T2, T3, and T4, respectively. The subjects’ psychosocial adjustment deteriorated after surgery. Results from generalized estimating equations indicated that financial status, cancer stage, pain, speech problems, social eating problems, and less sexuality were significant predictors of changes in psychosocial adjustment. Patients with insufficient income, stage III/IV cancer, severe pain, speech problems, social eating problems, and less sexuality were at higher risk for postoperative psychosocial maladjustment. Continued psychosocial assessment and appropriate supportive measures are needed to strengthen the psychosocial adjustment of these high-risk groups.

## 1. Introduction

Oral cancer is Taiwan’s fifth leading cause of cancer death [1]. Compared with the worldwide data, Taiwan has a much higher incidence and prevalence of oral cancer [2]. The age-standardized incidence rates per 100,000 people in 2020 were 30.6 and 3.06 for males and females, respectively. The age-standardized mortality rates per 100,000 people in 2020 were 11.48 and 0.95 for males and females, respectively [3]. The five-year relative survival rate was 56.4% and 65.1% for males and females, respectively [4].

Psychosocial adjustment to illness refers to managing intrapsychic and social demands in response to physical disease [5]. Such adjustments may include managing the impact of physical illness on the individual’s concerns about health, the work environment, family life, sexual function or relationships, relationships with extended family, social and leisure activities, and disturbing thoughts and feelings about the physical illness [5]. A reasonable psychosocial adjustment will help patients face the psychosocial challenges of oral cancer and its treatment. Oral cancer patients can become long-term survivors [6,7]. However, these survivors often face disease and treatment-related adverse effects, including facial disfigurement, dysphagia, xerostomia, and truisms [7]. These physical sequelae compromise patients’ body image, verbal communication, and social interactions and negatively impact patients’ psychosocial well-being [8]. Compared with other types of cancer, patients with oral cancers reported more psychosocial issues, including anxiety, depression, social isolation, and work and relationship problems [9]. Understating the factors that influence psychosocial adjustment in patients with oral cancer will help to identify risk groups for psychosocial maladjustment and develop interventions to strengthen patients’ psychosocial adjustment.

Facial disfigurement has been described as a state in which a person’s facial appearance has been medically severe and persistently damaged [10]. Despite advances in reconstructive surgery, oral cancer surgery can still lead to severe facial disfigurement depending on the location and stage of cancer. Facial disfigurement can negatively impact patients’ psychosocial well-being [11,12]. Appearance affects an individual’s body image and self-concept. Facial disfigurement makes contact with others difficult and embarrassing. The more concerned patients were about their facial deformities, the worse their body image, and the more likely they were to avoid social activities [12]. The social stigma of disfigurement can also bring psychological distress to the disfigured [13]. Hence, facial disfigurement may negatively affect the psychosocial adjustment of patients with oral cancer.

Patients with oral cancer experienced physical symptoms and side effects during and after treatment, with gradual remission over 3 to 12 months. Common symptoms in patients following oral cancer surgery include pain, dry mouth, sticky saliva, dental problems, and difficulty speaking, chewing, eating, and swallowing [14,15]. These symptoms can negatively impact a patient’s psychosocial adjustment. For example, difficulty with swallowing and chewing interferes with a patient’s eating, and the patient may avoid social eating to prevent embarrassment during eating. Difficulty speaking causes communication difficulties and interferes with social activities [16], affecting the patient’s workability.

Social support is the support individuals can obtain from their social network when needed [17]. Sources of social support include family, friends, neighbors, colleagues, caregivers, etc. It can take the form of emotional support (e.g., trust and caring), informational support (e.g., giving advice), instrumental support (e.g., direct material assistance), or appraisal support (e.g., affirmation) [17]. Social support is an essential resource for coping with stress and psychosocial adjustment. Past research has found that social support can help cancer patients manage psychological stress, reduce anxiety and depression, and improve their quality of life [18].

In summary, previous studies showed that oral cancer patients had poor psychosocial adjustment. Facial disfigurement, symptoms, and poor social support negatively impact oral cancer patients’ psychosocial adjustment. However, most of these findings were from cross-sectional studies. Few previous studies have investigated the changes in psychosocial adjustment over time in oral cancer patients. Oral cancer patients face different psychological and social challenges after reconstructive surgeries. Information on postoperative psychosocial adjustment changes and their influencing factors can help identify high-risk groups of maladjustment and develop appropriate measures to enhance psychosocial adjustment in patients with oral cancer.

Therefore, the study-specific aims were: (1) to describe the changes in psychosocial adjustment after oral cancer surgery; (2) to explore the effects of demographics, disease characteristics, facial disfigurement, social support, and symptoms on psychosocial adjustment.

## 2. Materials and Methods

### 2.1. Study Design and Subject Recruitment

This prospective longitudinal study was conducted from 2010 to 2013. Oral cancer patients who met the following eligibility criteria were recruited from the oral and maxillofacial surgical wards or the otolaryngology wards of two hospitals in Taiwan. One is a 3000-bed general hospital in Taipei, and the other is a 1000-bed general hospital in Hualien (eastern Taiwan). The inclusion criteria were (a) 20 years of age or older, (b) scheduled for reconstructive surgery for oral cancer, and (c) able to read Chinese. The exclusion criteria were: (a) with recurrence of oral cancer, (b) previously received reconstructive surgery for oral cancer, or (c) diagnosed with psychiatric illness. All subjects gave written informed consent before participating in the study.

### 2.2. Sample Size Determination

The required sample size was estimated using G-Power version 3.1 (Heinrich-Heine-Universität Düsseldorf, Düsseldorf, Germany). There have been no previous reports on the impacts of the study variables on postoperative psychosocial adjustment. The required sample size was estimated using the medium effect size suggested by Cohen [19]. The input parameters are an F-test, four repeated measures, a within-factor design, a correlation of 0.5 among repeated measures, a medium effect size (f = 0.25), a power of 80%, and a significance level of 0.05. A sample of 24 was required to analyze psychosocial adjustment changes over time. To explore potential predictors of psychosocial adjustment, an estimated 45 subjects were required. Given the longitudinal nature of the study, a dropout rate of 25% was estimated. Therefore, a sample of 62 subjects was recruited to overcome potential dropout issues. The final sample in the analysis included 50 patients.

### 2.3. Data Collection

Data collection occurred in each patient’s room (for baseline data) and a quiet room at outpatient clinics (for follow-up data). One of the investigators (Y.-W.C.) and a research assistant collected data from each subject using self-reported questionnaires. The research assistant with a bachelor’s degree in public health was trained in research protocols and data collection procedures. Subjects self-administered the study questionnaire. For subjects who had difficulty reading or comprehending the questionnaire, the data collector read each question to the subjects. Data on psychosocial adjustment, symptoms, and social support were collected before surgery (T1) and one month (T2), three months (T3), and five months after hospital discharge (T4). Data on demographics, disease variables, and facial disfigurement were collected at T1 only.

### 2.4. Instruments

In this study, demographics, disease characteristics, facial disfigurement, social support, and symptoms were the independent variables, and psychosocial adjustment was the dependent variable. Demographic data were collected from each subject, including age, gender, education level, marital status, employment status, and financial status. Disease characteristics, including cancer location, cancer stage, and adjuvant therapy, were collected from each subject’s medical records. Facial disfigurement was measured using a patient-rated facial disfigurement analogue scale [20]. Subjects rated the degree to which their facial appearance had changed due to the surgery on a visual analog scale of 0 (not at all) to 100 (worst possible). The scale has shown good psychometric properties in previous studies [11,20].

The following instruments were used to collect data for the study variables at all four data collection time points. Social support was measured using a social support scale developed in Chinese [21]. The scale has 16 items that measure four dimensions of social support: appraisal support, informational support, emotional support, and instrumental support. Subjects rated each item on a Likert scale (0 (never) to 3 (always)) to indicate how often they had received support from family members or essential others in the past month. The sum of all items is the social support score, with a possible range of 0–48. The higher the score, the greater the perceived support. The scale has shown good psychometric properties in previous studies on heart transplants [22], dialysis [23], and cancer patients [24]. In this study, Cronbach’s alpha for this scale was 0.94.

Symptoms were measured using the Chinese version of the European Organization for Research and Treatment of Cancer Quality of Life Questionnaire Head and Neck Cancer Module (EORTC QLQ-H&N35) [22,25]. The QLQ-H&N35 consists of 35 items, including 7 multi-item scales and 11 single-item scales. Seven multi-item scales were scored on a 4-point Likert scale (1 (not at all problem) to 4 (very much)) to assess pain, swallowing, taste and smell problems, speech problems, trouble with social eating, trouble with social contact, and less sexuality. Of the 11 single-item scales, 6 items used a 4-point Likert scale to assess teeth, opening the mouth, dry mouth, thick saliva, cough, and feeling unwell; 5 items were scored as yes or no to assess pain medication, nutritional supplements, feeding tube, weight loss, and weight gain. Only the 7 multi-item scales and the 6 single-item scales with a 4-point scale for scoring were used in this study. For all items and scales, high scores indicated more severe symptoms. Scores on all composite scales were calculated as the average of all items in these scales. These scores were then converted to normalized scores ranging from 0 to 100 using a linear transformation according to the scoring procedure. A high score indicates a higher level of symptomatology/problems. In previous studies, this scale has shown favorable psychometric properties in the HNC population [25]. In the current study, the internal consistency of the scale was acceptable, with Cronbach’s alpha coefficients of 0.70 (pain), 0.74 (swallowing), 0.61 (taste and smell problems), 0.81 (speech problems), 0.84 (social eating), 0.78 (social contact), and 0.85 (sexuality).

Psychosocial adjustment was measured using the Chinese version of the Psychosocial Adjustment to Illness Scale-Self Report (PAIS-SR) [26]. The 46-item scale measures seven major adjustment domains: healthcare orientation (8 items), vocational environment (6 items), domestic environment (8 items), sexual relationships (6 items), extended family relationships (5 items), social environment (6 items), and psychological distress (7 items). Each item was scored on a 4-point scale (0 (no problem) to 3 (a lot of difficulty)), with higher scores indicating worse adjustment. Scores for all domain scales were calculated as the total score of all items in those scales. These scores were then converted to standardized T-scores, with a possible range of 0–100 for each domain [27]. The score for PAIS-SR was calculated by summing the seven domain T-scores to provide global adjustment information, with a possible range of 0–700. A higher score indicates more difficulty experienced. A cutoff score of 393 was considered the clinical level of maladjustment [27]. In past studies involving cancer populations, the scale has shown acceptable reliability and validity [11,27]. In the current study, Cronbach’s alpha coefficients ranged from 0.52–0.87 on the seven domain scales and 0.91 on the full scale. The PAIS-SR score of all 46 items was used to represent the psychosocial adjustment for all analyses in this study.

### 2.5. Data Analysis

All statistical analyses were conducted using the Statistical Package for Social Sciences 20.0. Chi-square and Mann–Whitney tests were used to compare differences in demographics and disease characteristics between subjects who completed the study and those who were lost to follow-up. Descriptive statistics were used to describe study variables. A univariate generalized estimating equation (GEE) was used to analyze changes in psychosocial adjustment over time (from T1 to T4). Paired *t*-tests further explored differences in psychosocial adjustment scores between time points. A multivariate GEE with an exchangeable correlation structure was used to analyze the effects of demographics, disease variables, facial disfigurement, social support, and symptoms on psychosocial adaptation. The psychosocial adjustment was entered as the dependent variable. Time, demographics, disease variables, facial disfigurement, social support, and symptoms were entered as the independent variables.

## 3. Results

### 3.1. Subjects’ Characteristics

Seventy-nine potential subjects were approached. Five individuals did not meet the eligibility criteria, and 12 refused to participate. Sixty-two eligible individuals signed informed consent and participated in the study. At T2, eight subjects were lost to follow-up due to disease status (*n* = 2), failure to return or loss of contact (*n* = 2), and disinterest or inability to cooperate (*n* = 4). At T3, four subjects were lost to follow-up due to disease status (*n* = 1), failure to return or loss of contact (*n* = 2), and disinterest or inability to cooperate (*n* = 2). Fifty subjects completed the study, and their data were included in the analysis. Chi-square and Mann–Whitney tests showed no significant difference in demographics and disease characteristics between subjects who completed the study and those who were lost to follow-up.

We followed postoperative oral cancer patients for up to five months with an acceptable dropout rate of 19.4%. Most subjects were middle-aged men with high school education and poor financial status. Furthermore, 46% of subjects had buccal mucosa cancer, 36% had stage IV cancer, and 60% received adjuvant therapy (Table 1). This demographic and disease profile is similar to the epidemiological data for oral cancer in Taiwan [3].

### 3.2. Social Support

The mean (standard deviation, SD) support for T1, T2, T3, and T4 was 39.66 (9.43), 36. 9 (11.17), 36.35 (11.24), and 34.76 (10.67), respectively. Univariate GEE analysis results showed that social support had significant time effects (Table 2), indicating that both sources of support change significantly over time. Social support was highest at T1 and gradually decreased over time.

### 3.3. Symptoms

At T1, the most severe symptom experienced was teeth problems (median = 50, SD = 37.64). At T2, the most severe symptom was trouble with social eating (median = 54.2, interquartile range (IQR): 33.3–72.9). At T3, the most severe symptoms were trouble with social eating (median = 66.7, IQR: 33.3–75.0), teeth problems (median = 66.7, IQR: 33.3–100), opening the mouth (median = 66.7, IQR: 33.3–100), and sticky saliva (median = 66.7, IQR: 33.3–100). At T4, the three most severe symptoms were opening the mouth (median = 66.7, IQR: 33.3–100) and trouble with social eating (median = 50, IQR: 33.3–83.3, Table 2).

**Figure 1:**
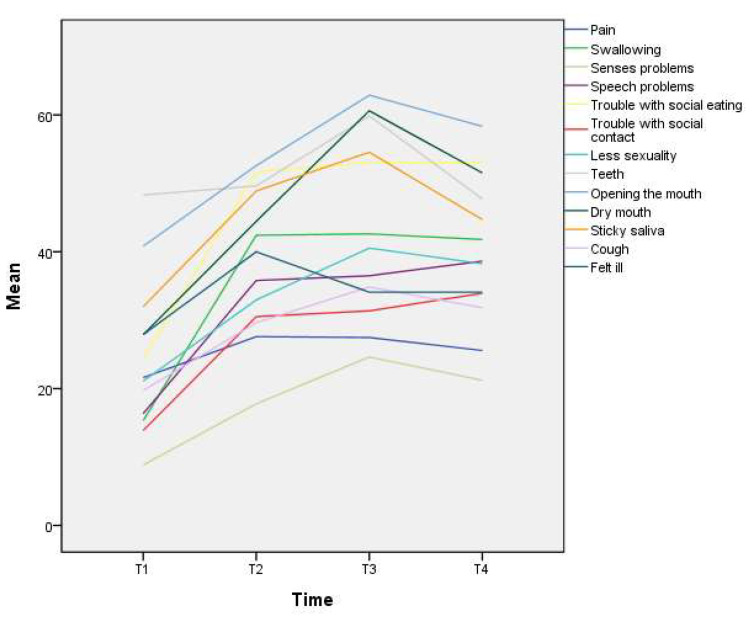
Oral cancer patients’ symptoms change over time.

Univariate GEE analysis showed that all symptoms, except the teeth problems, had a significant time effect, indicating that symptoms change significantly over time (Table 2). Most of these symptoms were aggravated after surgery, the most severe at T3, maintained or slightly improved from T3 to T4 (Figure 1).

### 3.4. Psychosocial Adjustment

**Figure 2:**
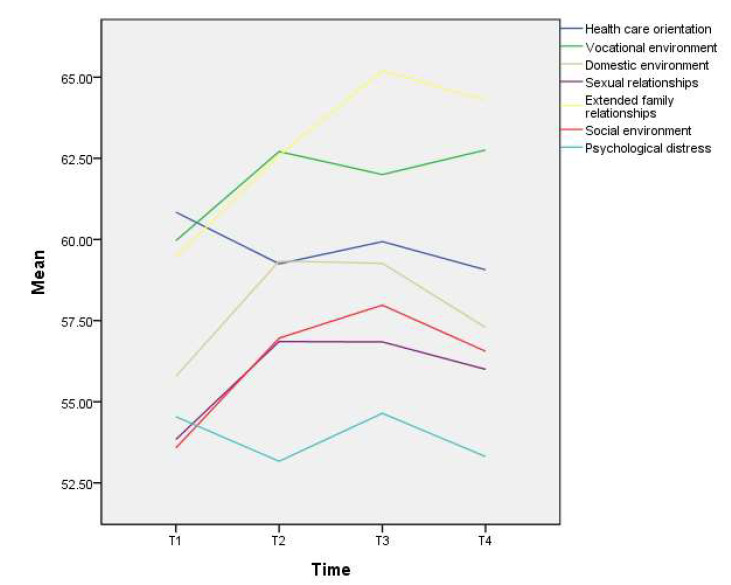
Oral cancer patients’ seven domains of psychosocial adjustment.

The mean (SD) PAIS-SR scores for T, T2, T3, and T4 were 398.0 (43.1), 410.9 (50.8), 415.9 (52.2), and 409.3 (52.4) (Table 2). Univariate GEE analysis results showed a significant time effect. Subjects’ PAIS-SR scores were significantly higher at T2 (unstandardized coefficient (B) = 12.8), T3 (B = 15.6), and T4 (B = 10.9) than at T1, indicating that subjects experienced more psychosocial adjustment challenges after surgery. Paired *t*-tests further explored differences in PAIS-SR scores between time points. The results showed a statistically significant difference between T1 and T2 (mean difference (MD) = 12.4, 95% confidence interval (CI): 1.7~23.0); between T3 and T4 (MD = −6.4, 95%CI: −12.3~−0.5). The difference between T2 and T3 was not statistically significant. These results suggest that subjects’ psychosocial adjustment challenges increased from T1 to T2, maintained from T2 to T3, and decreased from T3 to T4. Taking 393 as the cut-off point, 50%, 59.2%, 66%, and 62% of patients reported psychosocial maladjustment at T1, T2, T3, and T4, respectively. Of the seven domains of psychosocial adjustment, subjects experienced the most significant challenges in extended family relationships, vocational environment, and healthcare orientation (Figure 2).

### 3.5. Factors Associated with Psychosocial Adjustment

Results from GEE indicated that financial status, cancer stage, pain, speech problems, social eating problems, and decreased sexuality were significantly associated with psychosocial adjustment. Patients with enough income reported better psychosocial adjustment than patients without (B = −28.31, *p* = 0.041). Patients with stage I cancer had better psychosocial adjustment than patients with stage IV (B = 25.33, *p* = 0.047) and stage III cancers (B = 31.13, *p* = 0.026). Patients with severer pain (B = 0.38, *p* = 0.025), more speech problems (B = 0.39, *p* = 0.040), more trouble with social eating (B = 0.35, *p* = 0.013), and less sexuality (B = 0.25, *p* = 0.028) had worse psychosocial adjustment (Table 3). The following variables were not significantly associated with psychosocial adjustment: age, education level, marital status, employment status, tumor location, adjuvant therapy, facial disfigurement, social support, senses problems, trouble with social contact, teeth problems, opening the mouth, dry mouth, sticky saliva, cough, and felt ill.

## 4. Discussion

### 4.1. Psychosocial Adjustment

Our results show that patients with oral cancer had poor psychosocial adjustment. At each of the four data collection time points, more than half of the subjects had psychosocial maladjustment. Overall, the psychosocial adjustment of subjects worsened over time, and they reported the worst psychosocial adjustment three months after surgery. The findings suggest that oral cancer patients face significant psychosocial adjustment challenges after reconstructive surgery, especially in the first three months. Healthcare professionals should pay close attention to the psychosocial needs of this population and provide support to help patients cope with psychosocial challenges. Attention should be paid to adapting to changes in extended family relationships, vocational environment, and healthcare orientation, as subjects reported these were the most challenging areas.

### 4.2. Factors Associated with Psychosocial Adjustment

We found that financial status, cancer stages, pain, speech problems, social eating problems, and less sexuality were significant predictors of psychosocial adjustment after reconstructive surgery in patients with oral cancer. Like what was reported in cross-sectional studies, patients with not enough income [21], cancer stage III/ IV [28], severer pain [29], more speech problems [30], more social eating problems, and less sexuality report poorer psychosocial adjustment. Patients with these characteristics or symptoms are at higher risk for postoperative psychosocial maladjustment. These findings are interpreted in terms of *p*-values, which may not be clinically meaningful findings. However, the study’s finding provides preliminary data for understanding changes in psychosocial adjustment after oral cancer surgery and the potential impact of facial disfigurement, social support, and symptoms on psychosocial adjustment. Given the high prevalence and worsening of psychosocial maladjustment in this population over time, continued psychosocial assessment and appropriate supportive measures are needed to enhance psychosocial adjustment in these at-risk groups. Speech and social eating problems should be noted. Many oral cancer patients experience social avoidance due to concerns about speech or eating, which negatively impacts patients’ psychosocial adjustment [29]. In addition, postoperative oral cancer patients require ongoing assessment and appropriate interventions to help patients manage symptoms, especially during the three months following surgery. Particular attention should be paid to teeth problems, trouble with social eating, opening the mouth, and sticky saliva.

Unlike previous research reports [24], we found that psychosocial adjustment was independent of age, education, marital status, employment status, cancer location, and type of cancer treatment. Different patient populations and study designs may partially explain this discrepancy. Our subjects were recruited from two hospitals in Taiwan, and their characteristics may differ from patients in other clinical settings. Instead of a cross-sectional design, we used a longitudinal study design and followed patients with oral cancer for up to 5 months after discharge.

Furthermore, unlike previous research reports, we found no significant effect of facial disfigurement on psychosocial adjustment. Our results fail to support the research hypothesis that facial disfigurement negatively affects patients’ psychosocial adjustment over time. This difference may be because the perceived disfigurement of the face differs from the actual impairment of oral function, which may significantly impact psychosocial adjustment. Moreover, the effects of facial disfigurement on psychosocial adjustment may be primarily mediated by functional impairment or social isolation (e.g., speech or social eating problems) rather than direct effects [30,31]. Our findings suggest that patients with oral cancer experience many uncomfortable symptoms. Among them, pain, speech problems, social eating problems, and less sexuality significantly affected changes in psychosocial adjustment over time. The effects of other symptoms on psychosocial adjustments, such as sensory problems, social contact, teeth problems, opening the mouth, dry mouth, sticky saliva, cough, and felt ill, were not statistically significant.

### 4.3. Study Limitations

The study was limited by its small sample size, the use of a convenient sample, and the recruitment of male subjects only. We recruited a convenience sample of oral cancer patients from two medical centers in Taiwan. Characteristics of subjects may differ from patients in other clinical settings. Moreover, women may be more sensitive to facial changes. Although we did not exclude women, only men participated in this study. It is difficult to recruit a representative sample of female patients due to the low incidence of oral cancer in women. Therefore, our findings may not generalize beyond this sample. When considering the impact of perceived financial stress on psychosocial adjustment, we asked subjects to indicate how they viewed their income as meeting their needs, rather than their actual income. However, having enough or enough income can be subjective and vary from person to person. Using better scales could provide better information about subjects’ financial status.

## 5. Conclusions

Postoperative oral cancer patients experience poor psychosocial adjustment that worsens over time. Patients with poor financial status, cancer stage III/IV, severe pain, more speech problems, more social eating problems, and less sexuality were at the most significant risk for psychosocial maladjustment. Identifying these risk factors is essential because at-risk individuals can be targeted for early psychosocial assessment and intervention. Enhancing the psychosocial adjustment of oral cancer patients requires ongoing support and team-based and multidisciplinary collaborative care.

## Figures and Tables

**Table 1:** Demographics, disease characteristics, and perceived facial disfigurement (*N* = 50).

Variables	Frequency	%	Mean (SD)	Range
Hospital				
A	31	62		
B	19	38		
Age			50.04 (10.53)	32–78
Gender				
Male	50	100		
Education level				
Primary school and below	9	18		
Middle school	29	58		
College and above	12	24		
Marital status				
Single	16	32		
Married	34	68		
Employment status				
No	40	80		
Yes	9	18		
Financial status				
Not enough	22	44		
Enough	22	44		
More than enough	6	12		
Tumor Location				
Buccal mucosa	23	46		
Tongue, mouth floor	17	34		
Gingiva, lips	9	18		
Cancer stage				
I	9	18		
II	8	16		
III	14	28		
IV	18	36		
Adjuvant therapy				
No	20	40		
Yes	30	60		
Facial disfigurement			45.14 (32.25)	0–100

SD, standard deviation.

**Table 2:** Psychosocial adjustment, social support, and symptoms at pre-operation, and one month, three months, and five months after discharge (*n* = 50).

Variable	Time	Mean	SD	Range	B	*X^2^*	*p*-Value
Psychosocial adjustment	T4	409.3	52.4	275–487	10.9	4.5	0.033 *
	T3	415.9	52.2	275–490	15.6	9.3	0.002 **
	T2	410.9	50.8	301–515	12.8	6.5	0.011 *
	T1	398.0	43.1	309–486	0		
Social support	T4	34.8	10.7	0–48	−4.8	10.0	0.002 **
	T3	36.4	11.2	10–48	−3.0	3.9	0.048 *
	T2	36.9	11.2	2–48	−3.0	4.2	0.042 *
	T1	39.7	9.4	9–48	0		
		**Median**	**IQR**				
Pain	T4	25.0	8.3–33.3	0–75	4.2	2.0	0.161
	T3	33.3	12.5–37.5	0–75	6.1	4.1	0.042 *
	T2	25.0	8.3–33.3	0–91.67	5.9	4.1	0.043 *
	T1	16.7	8.3–27.1	0–66.67	0		
Swallowing	T4	41.7	20.8–58.3	0–100	26.2	49.6	<0.001 ***
	T3	41.7	25.0–70.8	0–100	28.3	57.7	<0.001 ***
	T2	33.3	16.7–66.7	0–100	26.6	53.4	<0.001 ***
	T1	8.3	0–25	0–50	0		
Senses problems	T4	16.7	0–33.3	0–100	12.6	8.5	0.004 **
	T3	16.7	0–50	0–66.67	16.1	13.8	<0.001 ***
	T2	0	0–33.3	0–100	9.2	5.1	0.024 *
	T1	0	0–0	0–100	0		
Speech problems	T4	33.3	22.2–61.1	0–77.78	22.0	37.2	<0.001***
	T3	33.3	16.7–55.6	0–88.89	20.1	31.0	<0.001 ***
	T2	33.3	11.1–55.6	0–100	19.2	29.3	<0.001 ***
	T1	11.1	0–22.2	0–100	0		
Trouble with social eating	T4	50.0	33.3–83.3	0–100	28.3	53.1	<0.001 ***
	T3	66.7	33.3–75.0	0–100	29.7	58.5	<0.001 ***
	T2	54.2	33.3–72.9	0–100	27.7	52.8	<0.001 ***
	T1	16.7	0–41.7	0–100	0		
Trouble with social contact	T4	33.3	16.7–40.0	0–100	19.5	33.8	<0.001 ***
	T3	33.3	16.7–40.0	0–100	16.9	25.5	<0.001 ***
	T2	26.7	6.7–46.7	0–100	16.9	26.4	<0.001 ***
	T1	6.7	0–26.7	0–73.33	0		
Less sexuality	T4	33.3	16.7–50.0	0–100	17.4	13.6	<0.001 ***
	T3	33.3	0–66.7	0–100	19.8	17.8	<0.001 ***
	T2	33.3	16.7–33.3	0–100	13.5	8.6	0.003 **
	T1	16.7	0–33.3	0–100	0		
Teeth problems	T4	33.3	33.3–66.7	0–100	−2.1	0.13	0.720
	T3	66.7	33.3–100	0–100	11.3	3.8	0.052
	T2	33.3	33.3–100	0–100	−0.7	0.0	0.904
	T1	50	0–75	0–100	0		
Opening the mouth	T4	66.7	33.3–100	0–100	17.0	11.9	0.001 **
	T3	66.7	33.3–100	0–100	20.4	17.2	<0.001 ***
	T2	33.3	33.3–100	0–100	13.2	7.5	0.006 **
	T1	33.3	0–66.7	0–100	0		
Dry mouth	T4	33.3	33.3–100	0–100	22.7	20.5	<0.001 ***
	T3	33.3	33.3–100	0–100	31.4	39.1	<0.001 ***
	T2	33.3	33.3–66.7	0–100	17.3	12.3	<0.001 ***
	T1	33.3	0–33.3	0–100	0		
Sticky saliva	T4	33.3	33.3–66.7	0–100	11.9	5.7	0.017 *
	T3	66.7	33.3–100	0–100	22.6	20.5	<0.001 ***
	T2	33.3	33.3–66.7	0–100	16.6	11.5	0.001 **
	T1	33.3	0–33.3	0–100	0		
Cough	T4	33.3	0–33.3	0–100	12.1	11.2	0.001 **
	T3	33.3	33.3–33.3	0–100	15.9	19.4	<0.001 ***
	T2	33.3	0–33.3	0–100	10.2	8.2	0.004 **
	T1	33.3	0–33.3	0–100	0		
Felt ill	T4	33.3	33.3–33.3	0–100	5.7	1.6	0.202
	T3	33.3	33.3–33.3	0–100	6.0	1.8	0.180
	T2	33.3	33.3–66.7	0–100	11.7	7.0	0.008 **
	T1	33.3	0–33.3	0–100	0		

Note. Generalized estimating equations for repeated measurements and an exchangeable correlation structure were used. T1, preoperative; T2, one month after discharge; T3, three months after discharge; T4, five months after discharge; SD, standard division; IQR, interquartile range; B, unstandardized coefficient; X^2^, value of Wald chi-square; * *p* < 0.05; ** *p* < 0.01; *** *p* < 0.001.

**Table 3:** Factors associated with psychosocial adjustment (*n* = 50).

Variables	*B*	SE	95% CI		Wald *X^2^*	*p*-Value
			Lower	Upper		
Intercept	395.75	29.72	337.50	454.00	177.32	<0.001 ***
Time						
T4 vs. T1	−10.52	7.36	−24.94	3.91	2.04	0.153
T3 vs. T1	−8.83	7.28	−23.1	5.44	1.47	0.225
T2 vs. T1	−6.24	6.60	−19.18	6.69	0.90	0.344
Age	−0.20	0.44	−1.07	0.67	0.21	0.648
Hospital (B vs. A)	−14.79	10.07	−34.52	4.95	2.16	0.142
Education level						
College vs. Primary school	−0.30	15.16	−30.02	29.4	0.00	0.984
Middle vs. Primary school	2.62	11.84	−20.58	25.82	0.05	0.825
Marital (Married vs. Single)	−5.29	9.51	−23.93	13.35	0.31	0.578
Employment (Yes vs. No)	−20.84	13.52	−47.33	5.65	2.38	0.123
Financial status						
More than enough vs. Not enough	−28.31	13.88	−55.51	−1.11	4.16	0.041 *
Enough vs. Not enough	−3.70	8.86	−21.06	13.66	0.17	0.676
Tumor Location						
Gingiva, lips vs. Buccal	19.77	10.77	−1.35	40.88	3.37	0.067
Tongue, mouth floor vs. Buccal mucosa	−5.28	9.65	−24.18	13.63	0.30	0.584
Cancer stage						
IV vs. I	25.33	12.76	0.33	50.33	3.94	0.047 *
III vs. I	31.12	13.96	3.76	58.49	4.97	0.026 *
II vs. I	13.72	11.60	−9.03	36.46	1.4	0.237
Adjuvant therapy (yes vs. no)	−14.94	8.98	−32.53	2.66	1.78	0.182
Facial disfigurement	0.19	0.13	−0.76	0.45	1.94	0.164
Social support	−0.29	0.33	−0.93	0.36	0.76	0.382
Pain	0.38	0.17	0.05	0.70	5.02	0.025 *
Swallowing	0.16	0.15	−0.14	0.46	1.07	0.301
Senses problems	0.15	0.13	−0.11	0.40	1.29	0.257
Speech problems	0.39	0.19	−0.75	−0.02	4.23	0.040 *
Trouble with social eating	0.35	0.14	0.07	0.63	6.17	0.013 *
Trouble with social contact	0.34	0.19	−0.03	0.70	3.28	0.070
Less sexuality	0.25	0.11	0.03	0.47	4.85	0.028 *
Teeth problems	0.04	0.08	−0.12	0.20	0.21	0.645
Opening the mouth	−0.13	0.09	−0.31	0.06	1.76	0.185
Dry mouth	0.02	0.10	−0.18	0.22	0.02	0.879
Sticky saliva	−0.12	0.11	−0.33	0.09	1.19	0.275
Cough	0.19	0.12	−0.05	0.42	2.42	0.119
Felt ill	0.16	0.13	−0.1	0.41	1.48	0.223

Note. Generalized estimating equations for repeated measurements and an exchangeable correlation structure were used. T1, preoperative; T2, one month after discharge; T3, three months after discharge; T4, five months after discharge. B, unstandardized beta; SE, standard error; X^2^, value of Wald chi-square; * *p* < 0.05, ***, *p* < 0.001.

## Data Availability

Data will be available from the corresponding author upon reasonable request.
